# Modulation of biochemical and physiological parameters in *Hordeum vulgare* L. seedlings under the influence of benzyl-butyl phthalate

**DOI:** 10.7717/peerj.6742

**Published:** 2019-04-19

**Authors:** Arpna Kumari, Rajinder Kaur

**Affiliations:** Department of Botanical and Environmental Sciences, Guru Nanak Dev University, Amritsar, Punjab, India

**Keywords:** Benzyl-butyl phthalate, Biochemical responses, Polyphenols profiling, Confocal microscopy, Scanning electron microscopy

## Abstract

**Background:**

Phthalates are man-made chemical compounds with numerous applications especially known for their use as plasticizers. They have weak bonding to the polymeric matrix or products in which they are used. Owing to this reason, they are readily released into the environment which makes them ubiquitous. The agricultural soils are also reported to be polluted with phthalates up to a considerable extent which causes adverse effects on flora and fauna. A few studies have been conducted on phthalate-induced phytotoxicity, which has revealed that phthalates affect the quality and yield of edible plants. In the last decades, some crops were analyzed for phthalate-induced adversities; among them, barley was the least explored.

**Methods:**

The present study has investigated the impact of benzyl-butyl phthalate (BBP) on barley (*Hordeum vulgare* L.) seedlings to address the biochemical, physiological consequences, and toxicological implications. After the exogenous exposure of BBP (*viz*. 0, 25, 50, 100, 200, 400, 800, 1,600 mg/L) for 7 days, barley seedlings were analyzed for different indices.

**Results:**

The exposure of BBP mediated a significant (*p* ≤ 0.05, 0.01) overall elevation in the contents of pigment, proline, soluble protein, carbohydrate, hydrogen peroxide (H_2_O_2_), and malondialdehyde (MDA) in shoots and roots of barley seedlings. The activities of superoxide dismutase (SOD), guaiacol peroxidase (POD), catalase (CAT), ascorbate peroxidase (APX), and glutathione reductase (GR) were also stimulated significantly in shoots and roots of seedlings against BBP stress except for SOD activity which declined in the roots. The polyphenols (non-enzymatic antioxidants) content was also altered in all the treated concentrations as compared to the control. Furthermore, BBP caused stomatal abnormalities, induced cytotoxicity, and loss of plasma membrane integrity.

**Conclusions:**

BBP disturbed the normal physiology of barley which could also affect the yield of the crop under field conditions.

## Introduction

Phthalate esters (PEs) are well-known additives or plasticizers for plastics, especially polyvinyl chloride (PVC) polymer used to inculcate elasticity, plasticity, and overall sustainability benefits (Mackintosh et al., 2004). The finished plastic products may contain up to 80% of PEs by weight (Sun, Wu & Gan, 2015). The annual world production of PEs was estimated to be approximately 2 million tons in the 1980s by the United States Environmental Protection Agency (USEPA), while in 2014 it was estimated to be 6 million tons per year (Millar & Hannay, 1986; Niu et al., 2014). There has been a threefold increase in PEs production in the last three decades, which is still increasing day by day and posing risk to the well-being of living organisms. PEs are prone to leach into the surroundings because of weak bonding forces with the plastics or polymers (Li et al., 2004). PEs are reported to act as potential endocrine disruptors, carcinogens, mutagens, teratogens, and reproductive toxicants (Larsson, Thuran & Gahnstrom, 1986; Amin et al., 2018). In humans, PEs have been reported to induce anti-androgenic effects on the fetus during early pregnancy; prenatal exposure was observed to be associated with shorter gestation age and various diseases like cardiovascular disease, type-2-diabetes, and hypertension (Whyatt et al., 2009; Bai et al., 2017). The main exposure routes of phthalates to humans are dermal contact, cosmetics, personal care products, packed food material, and fruits or vegetable grown in PAs contaminated soil. PEs are lipophilic in nature and readily adsorb on soils or sediments. From soils, plants are reported to accumulate PEs to a considerable extent and simultaneously it has raised food safety issues (Song & Hu, 2010). To sustain agricultural production, various technologies are introduced to traditional agricultural practices. Plasticulture is one such technology which is based upon the use of films, irrigation tubings, nursery pots, silage bags, and in a broader sense it is technology which makes the use of all kinds of plastic coverings. For instance, examples include plastic mulch films, row coverings, high and low tunnels, plastic greenhouses, etc. (https://en.wikipedia.org/wiki/Plasticulture). Most literature in existence has addressed PVC plastic films as a main contributing factor to phthalates pollution in soils (Wang et al., 2013; Gao & Wen, 2016). Use of wastewater or reclaimed water due to the scarcity of water in arid and semi-arid areas has also been proven as a significant source of phthalate pollution (Lesser et al., 2018). Furthermore, the other sources included sewage sludge used for improving the properties of soils, the release of industrial effluents, use of pesticides and fertilizers, and leaching from plastic waste (Cai et al., 2007; Guo & Kannan, 2012). In edible plants, the exposure of phthalates has unequivocally affected the growth, germination, and physiology of edible plants (Liao, Yen & Wang, 2009; Cheng & Cheng, 2012; Zhang et al., 2015; Kaur et al., 2017; Kumari & Kaur, 2017; Kumari & Kaur, 2018).

BBP has inescapable applications in the manufacturing of many products such as in vinyl foams, artificial leather, conveyor belts, traffic cones, etc (https://en.wikipedia.org/wiki/Benzyl_butyl_phthalate, 2019). It is one of the phthalates which are classified as priority environmental pollutants by the United States Environmental Protection Agency. It is one of the chemicals which cause reproductive toxicity and cancer as listed in California’s Proposition 65 (Safe Drinking Water and Toxic Enforcement Act of 1986). BBP is reported to cause various adverse effects on animals as well as humans, but the reports on the effects of BBP on edible plants are scarce in the literature. Furthermore, the detailed studies are still lacking on physiological consequences induced by BBP in barley i.e., an important crop plant after wheat, rice, and maize. Therefore, this study has been designed to envisage BBP-induced toxicological consequences in barley seedlings.

## Material and Methods

### Raising of barley seedlings

*Hordeum vulgare* var. VLB-118 seeds were procured from the Hill Agricultural Research and Extension Centre (HAREC) Bajaura, Kullu, Himachal Pradesh, India. BBP (CAS: 85-68-7, purity: 98%) was purchased from Himedia Laboratories Private Limited (India) and other chemicals used were of analytical grade. The stock solution of BBP (1,600 mg/L) was prepared using the method of Yin et al. (2003) with slight modifications. The required volume of BBP to form the stock solution was taken, diluted with ethanol and appropriate volume of Tween-20 and double distilled water (DDW) was added. Tween-20 was added and mixed thoroughly to ensure the homogeneous dispersion of BBP (Chen et al., 2011). The working solutions were prepared via serial dilution of stock solution (Kumari & Kaur, 2018).

In brief, for raising seedlings, barley seeds were surface sterilized using 0.01% mercuric chloride, presoaked in DDW and kept in Petri plates lined with Whatman filter paper no. 1. Twenty seeds were raised in closed Petri plates in triplicate. The seeds were moistened with different concentrations of BBP on alternate days and were germinated under light/dark regime of 16/8 h (Kumari & Kaur, 2018). The experiment was carried out under controlled conditions where temperature, humidity, and light intensity were set at 25 ± 0.5 °C, 75–80%, and 115 µmol m^2^s^−1^ respectively. The barley seedlings were harvested on the 7th day and preserved at −80 °C till the analysis of different parameters.

### Estimation of pigments content

For estimation of pigments, samples were extracted using 80% acetone. The optical density of acetone extract was observed at 470, 645, and 663 nm. For determination of photosynthetic pigments, equations given by Arnon (1949) were used, while carotenoids content was determined using the equation given by Lichtenthaler & Wellburn (1983). Furthermore, the ratios of chl a/chl b and total chl/carotenoids were calculated according to Cheng & Cheng (2012).

### Estimation of osmolytes

#### Proline content

The content of proline was estimated using the method proposed by Bates, Waldren & Teare (1973). The samples were homogenized in aqueous sulfosalicylic acid (3.0%) and the homogenate was filtered through Whatman filter paper. The reaction mixture consisted of the filtrate, acid-ninhydrin, and glacial acetic acid. The reaction mixture was heated for 1 h at 100 °C and reaction was terminated by cooling the test tubes in an ice-bath. To the resultant mixture, toluene was added followed by vigorous mixing which resulted in the formation of rusty red colored chromophore. The absorbance of chromophore was observed at 520 nm. L-proline was used as a standard.

#### Soluble proteins content

The protein content of seedlings was estimated using the Bradford method (Bradford, 1976). The samples were homogenized in potassium phosphate buffer (pH = 7.0) and were centrifuged at 12,000 rpm for 20 min. at 4 °C temperature. The Bradford reagent was added to the resultant solution and absorbance was recorded at 595 nm. Bovine serum albumin (BSA) was used as a standard.

#### Carbohydrate content

For estimation of carbohydrate content, anthrone reagent method was used (Yemm & Willis, 1954). The samples were hydrolyzed with 2.5 N hydrochloric acid (HCl) in the boiling water bath for 3 h. The solution was neutralized with sodium carbonate (Na_2_CO_3_) until the effervescence ceased and the volume was made upto 100 mL using DDW and was then centrifuged. Anthrone reagent was added to the supernatant and kept in the boiling water bath for 8 min. After cooling, the absorbance was observed at 630 nm. The carbohydrate content was estimated using glucose as a standard.

### Determination of oxidative stress

#### Hydrogen peroxide (H_2_O_2_) content

H_2_O_2_ is considered as an important indicator of oxidative stress. H_2_O_2_ content was determined using the method of Alexieva et al. (2001). The samples were extracted using 0.1% trichloroacetic acid (TCA). The reaction mixture was prepared by mixing leaf extract, 100 mM potassium phosphate buffer and potassium iodide (1.0 M). The reaction mixture was kept in darkness for 1 h. In this reaction, H_2_O_2_ present in leaf extract reacts with KI. The iodide ions are slowly oxidized to iodine (I_2_) that further reacts with iodide ions to form triiodide which can be visualized by the production of yellow color in solution. Thus, H_2_O_2_ is quantified by recording the absorbance of the reaction mixture at 390 nm using a spectrophotometer. The hydrogen peroxide content in the samples was determined using H_2_O_2_ as standard.

#### Malondialdehyde (MDA) content

MDA content was determined using the method of Heath & Packer (1968). The sample was homogenized in 0.1% TCA and centrifuged at 10,000 rpm for 5 min. To the supernatant, 0.5% thiobarbituric acid solution (prepared in 20% TCA) was added and followed by heating in the water bath at 95 °C for 30 min. After cooling, the absorbance was recorded at 532 and 600 nm.

### Extraction and analysis of antioxidative enzymes

The frozen fresh seedlings were used for the estimation of antioxidant enzymatic activities. The seedlings were pulverized in liquid nitrogen and homogenized in chilled 0.1 M potassium phosphate buffer (pH = 7.0). The homogenate was centrifuged for 20 min. at 12,000 rpm and 4 °C temperature. The supernatant was collected and used for the analysis of activities of different antioxidative enzymes as follows:

i. The activity of SOD (EC 1.15.1.1) was determined by the method given by Kono, Takahashi & Asada (1979). For the estimation of the activity, the reaction mixture was prepared which consisted of sodium carbonate buffer (50 mM, pH = 10.2), nitroblue tetrazolium (NBT, 96 µM), Triton X-100 (0.6%), hydroxylamine hydrochloride (20 mM, pH = 6.0), and enzyme extract. The activity of SOD was observed as a decrease in absorbance at 560 nm.
ii. POD (EC 1.11.1.7) activity was determined by the method given by Putter (1974). The reaction mixture contained potassium phosphate buffer (0.1 M, pH = 7.0), guaiacol (20 mM), H_2_O_2_ (12.3 mM), and enzyme extract. The rate of formation of tetra-guaiacol was measured at 436 nm and enzymatic activity was calculated using the extinction coefficient (25.5 mM^−1^cm^1^).
iii. CAT (EC 1.11.1.6) activity was determined using the method given by Aebi (1984). It is based on the initial disappearance rate of H_2_O_2_. The reaction was initiated using potassium phosphate buffer (0.1 M, pH = 7.0), H_2_O_2_ (150 mM), and enzyme extract. The decrease in absorbance was recorded at 240 nm. The enzyme activity was determined using the extinction coefficient (39.4 mM^−1^cm^−1^).
iv. APX (EC 1.11.1.11) activity was observed using the method of Nakano & Asada (1981). The reaction solution consisted of phosphate buffer (0.1 M, pH = 7.0), ascorbate (5 mM), H_2_O_2_ (0.5 mM), and enzyme extract. The decrease in absorbance was measured at 290 nm. The activity of the enzyme was determined using the extinction coefficient (2.8 mM^−1^cm^−1^).
v. The activity of GR (EC 1.8.1.7) was analyzed using the method of Carlberg & Mannervik (1985). The reaction was initiated with the addition of potassium phosphate buffer (0.05 M, pH = 7.6), ethylenediaminetetraacetic acid (EDTA, 3 mM), reduced nicotinamide adenine dinucleotide phosphate (NADPH, 0.1 mM), oxidized glutathione (GSSG, 1 mM), and enzyme extract. The absorbance was recorded at 340 nm. The enzymatic activity was calculated using the extinction coefficient (6.22 mM^−1^cm^−1^).

### Polyphenols profiling

The methanolic extract of seedlings was prepared and dissolved in methanol, filtered through 0.22 µm filter membranes and then, analyzed for different polyphenols using 130 MPa Shimadzu ultra high-performance liquid chromatography (UHPLC) system (NEXERA) purchased from Shimadzu Corporation (Kyoto, Japan). The system was coupled with degasser, solvent mixing unit, autosampler, column oven, photo-diode array detector, and system controller. The mobile phase consisted of solvent A: 0.01% acetic acid in water, and solvent B: 100% methanol. The analysis of results was performed using Lab Solutions Software.

### Cell membrane integrity and cytotoxic studies

The cell viability of treated root samples was observed using the method of Sasaki et al. (1997) with modifications. The samples were rinsed with phosphate buffer saline (PBS) followed by staining with propidium iodide (PI) for 30 min. in dark at room temperature. After staining, the root samples were washed in PBS for 3–5 times to remove the unbound stain particles from the root surface. The samples were scanned for confocal fluorescent imaging on Nikon A1R laser scanning confocal microscope (Nikon Corp., Japan) coupled with built-in Nikon NIS Element AR software for the acquisition of confocal images. He-Ne gas laser was used to excite the electrons at a wavelength of 535 nm.

### Stomatal morphological studies using scanning electron microscopy (SEM)

BBP-induced stomatal abnormalities were observed using the method of Liu et al. (2000) with modifications. The segment (4 or 5 mm) of seedling was cut followed by fixation in 2.5% glutaraldehyde for at least 24 h. Then, the samples were dehydrated in a graded ethanol series. The dehydrated samples were mounted on the stubs with double sided tape, air-dried, coated with silver, and the abaxial surface was observed under SEM (Carl Zeiss Evo LS 10, purchased from Carl Zeiss NTS, Oberkochen, Germany).

### Statistical analysis

The results were analyzed for mean, standard error, one-way, and two-way analysis of variance (ANOVA). All the experiments were performed in triplicate. Pearson’s bivariate correlation among different biochemical indices of shoots and roots was observed using SPSS 16.0 version (SPPS Inc., Chicago, IL, USA).

## Results

### Effect on pigments

**Figure 1:**
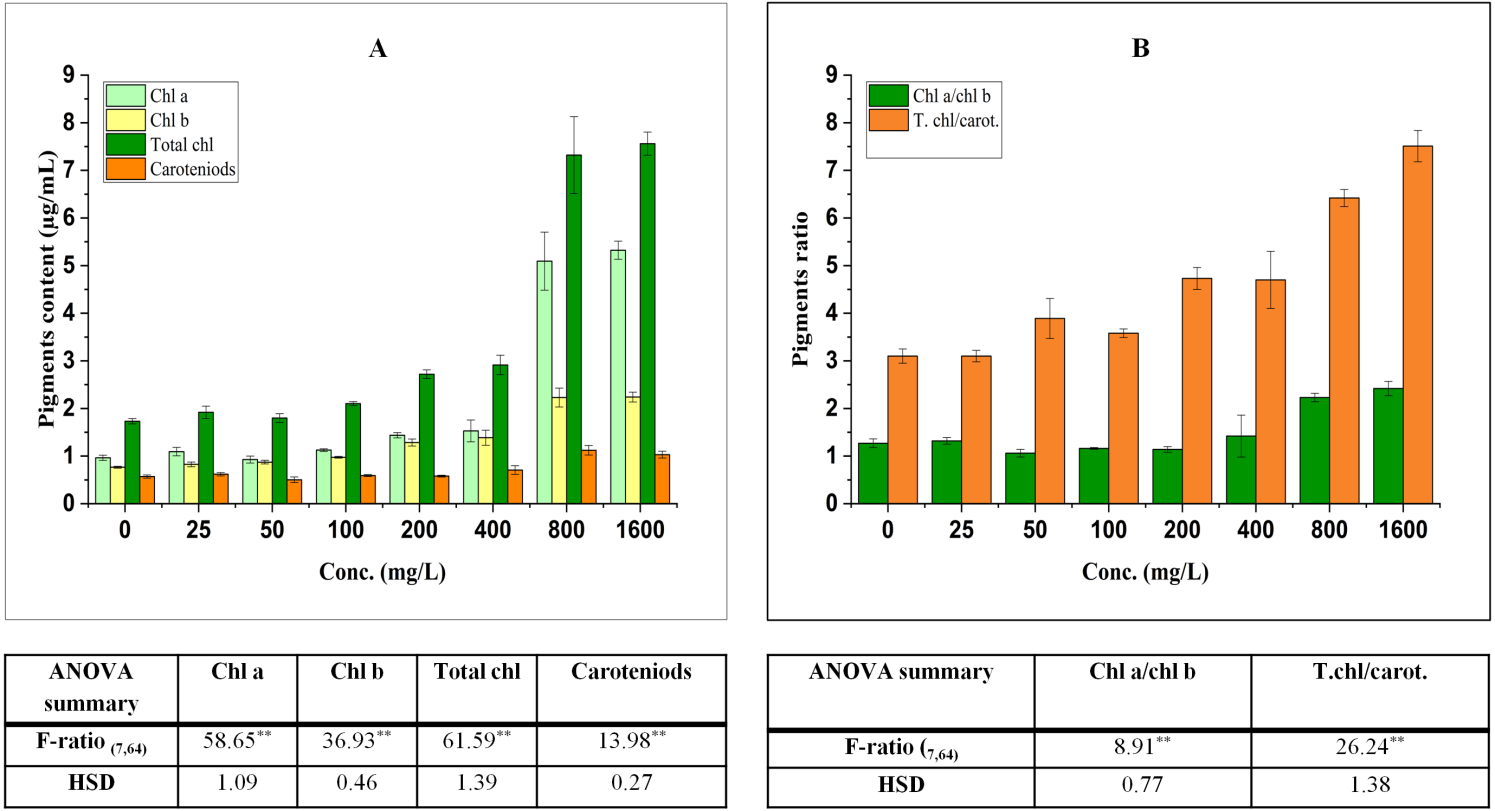
Effect of BBP on (A) pigments and (B) pigment ratios. Results are presented as mean ± S.E., *n* = 9. **Significant at *p* ≤ 0.01.

The effect on pigments is a well-known index to access the impact of different pollutants on plants because of their important roles in different metabolic processes. They have also been reported to affect photosynthetic potential and productivity (Carter, 1998). The effect of BBP on pigments has been shown in Fig. 1.

### Effect on chlorophyll content

The contents of pigments were significantly (*p* ≤ 0.05, 0.01) elevated in treated seedlings. The increase in chl a was concentration dependent (except at 50 mg/L) and percent increase was 13.42, 16.78, 49.00, 58.53, 428.23, 452.04% at 25, 100, 200, 400, 800, 1,600 mg/L respectively in comparison to the control. For the content of chl b, a significant increase was observed which was concentration dependent and percent increase ranged from 7.50 to 192.06% with respect to control. Similarly, the content of total chl was enhanced significantly and percent increase was 10.91, 3.95, 21.37, 57.17, 68.40, 323.05, 336.91% with respect to the control at 25, 50, 100, 200, 400, 800, 1,600 mg/L.

### Effect on carotenoids content

Carotenoids (accessory pigments) play an important role in carbon fixation process and also act as non-enzymatic antioxidants. In the present work, there was an overall significant (*p* ≤ 0.05, 0.01) increasing trend in the content of carotenoids which ranged from 1.89 to 97.11% as compared to the control.

The ratio of chl a to chl b was significantly (*p* ≤ 0.05, 0.01) modified by BBP as compared to the control. The ratio initially increased at 25 mg/L, while it was decreased from 50 to 200 mg/L then, again followed by an increase at higher concentrations (400–1,600 mg/L). The percent increase was ranged from 4.10 to 90.72% as compared to the control. The ratio of total chl to carotenoids followed a significant (*p* ≤ 0.05, 0.01) increasing trend except at 25 mg/L of BBP. The percent increase ranged from 15.37 to 142.11% as compared to the control.

### Effect on osmolytes

**Figure 2:**
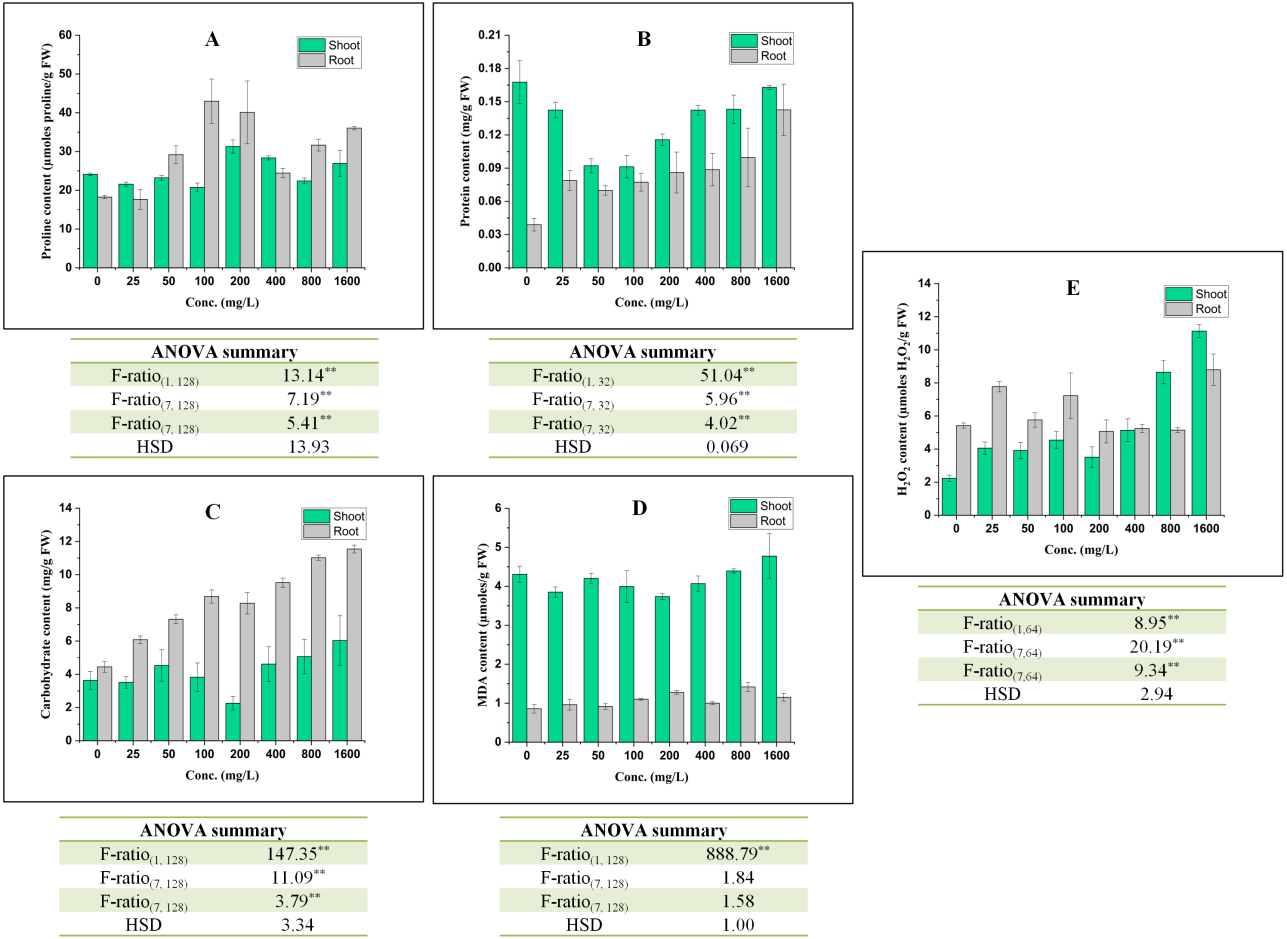
Effect of BBP on (A) Proline content, (B) Protein content, (C) Carbohydrate content, (D) MDA content, and (E) H_2_O_2_ content. Results are presented as mean ± S.E., *n* = 9 except protein content where *n* = 3. **Significant at *p* ≤ 0.01.

The effect of BBP on contents of osmolyte have been shown in Fig. 2. The proline content was observed to decrease significantly (*p* ≤ 0.05, 0.01) at lower concentrations (0–100 mg/L) but increased at higher concentration (except 800 mg/L) in the case of shoots. In roots, initially at 25 mg/L, proline content was decreased followed by a significant (*p* ≤ 0.05, 0.01) increase and percent increase ranged from 33.89 to 153.47% as compared to the control. This increase in proline content was not concentration dependent but more than control. In shoots, there was a significant (*p* ≤ 0.05, 0.01) decrease in the content of protein and percent decrease ranged from 2.80 to 45.80% as compared to the control. On other hand, in roots, the content of protein was observed to increase significantly (*p* ≤ 0.05, 0.01) with the increase in concentration except at 50 and 100 mg/L. The content of carbohydrate was observed to increase significantly (*p* ≤ 0.05, 0.01) in both shoots and roots. In shoots, carbohydrate content was observed to increase except at 25 and 200 mg/L. The percent increase ranged from 5.27 to 66.06% as compared to the control. In roots, the percent increase ranged from 36.70 to 159.36%, which is more apparent than in shoots.

### Effect on symptomatic indices of oxidative stress

In shoots, the content of MDA decreased significantly (*p* ≤ 0.05, 0.01) at 0–400 mg/L followed by an increase at 800 and 1,600 mg/L in comparison to the control. However, in roots, there was a significant (*p* ≤ 0.05, 0.01) increase in MDA content and the percent increase ranged from 6.77 to 65.16% with respect to the control.

The content of H_2_O_2_ was observed to increase significantly (*p* ≤ 0.05, 0.01) in both shoots and roots. In shoots, the increasing trend was concentrations dependent except at 25 and 200 mg/L. In roots, the maximum increase (62.30%) was observed at 1,600 mg/L.

### Effect on the activities of antioxidative enzymes

**Figure 3:**
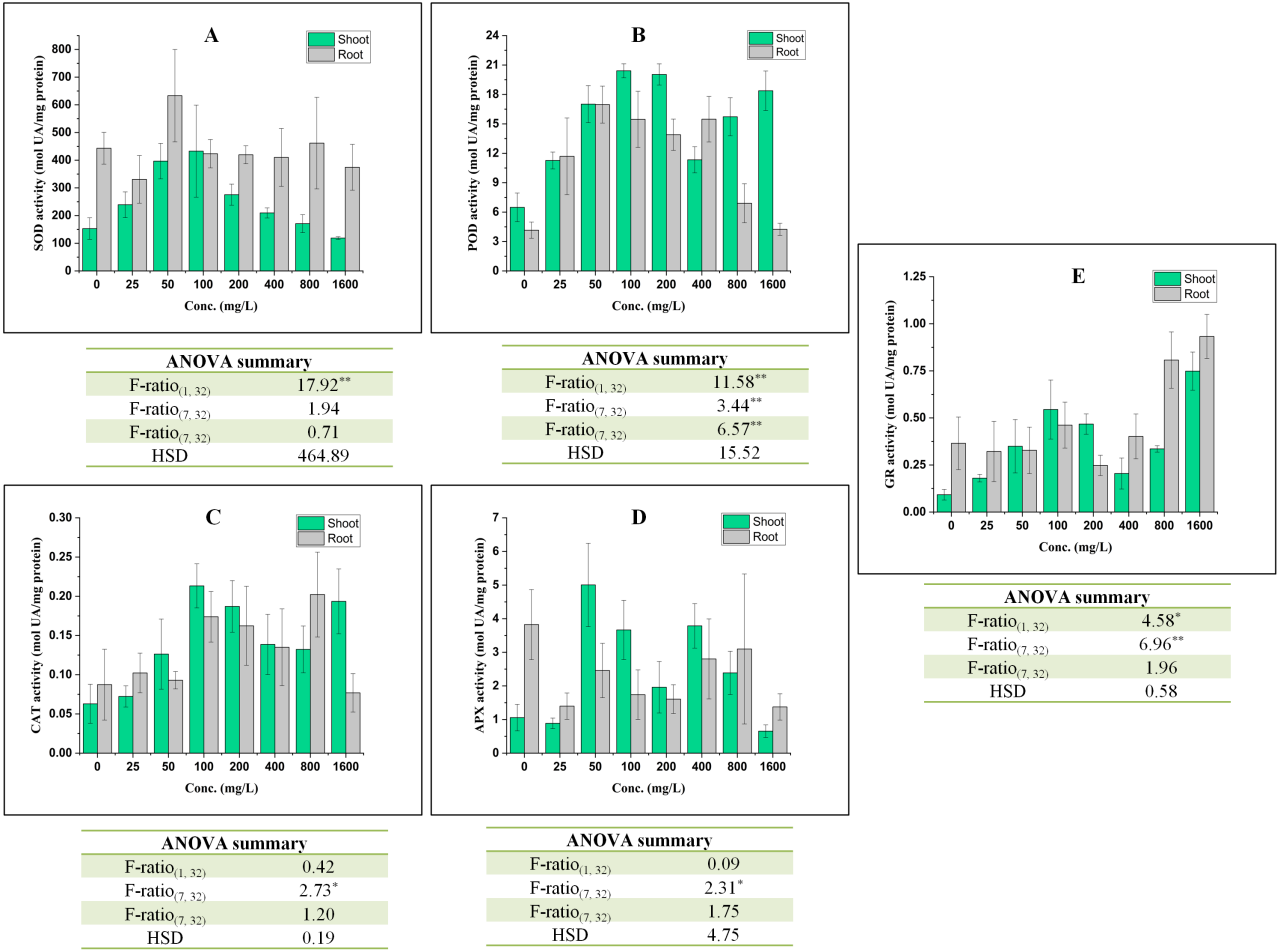
Effect of BBP on the activities of antioxidative enzymes (A) SOD, (B) POD, (C) CAT, (D) APX, and (E) GR. Results are presented as mean ± S.E., *n* = 3. **Significant at *p* ≤ 0.01, *significant at *p* ≤ 0.05.

Under stress, plants efficiently respond to elevated reactive oxygen species (ROS) via their antioxidative defense system which is one of the acclimatization strategies. In such conditions, two compartments *viz*. apoplast and chloroplast are of primary interest (Heath & Taylor, 1997) because these are the principal sites of ROS generation. Therefore, in this section, the responses of antioxidative defense system are discussed as follows (Fig. 3):

The activity of SOD was increased significantly (*p* ≤ 0.05, 0.01) in shoots except at 1,600 mg/L and percent increase ranged from 11.62 to 182.80% as compared to control. In roots, the activity of SOD was observed to alter significantly (*p* ≤ 0.05, 0.01) in all concentrations as compared to the control. The overall trend was decrease in the activity of SOD except at 50 and 800 mg/L of BBP.

In shoots, the activity of POD was recorded to increase significantly (*p* ≤ 0.05, 0.01) and percent increase ranged from 73.75 to 214.79% as compared to the control. The maximum increase was observed at 100 mg/L of BBP. In roots, the activity was also elevated like in shoots. The percent increase was ranged from 2.05 to 307.68% as compared to the control.

The activity of CAT was increased significantly (*p* ≤ 0.05) in both shoots and roots after the 7 days treatment but the effect of BBP was not significant on CAT activity. In shoots, the percent increase ranged from 14.95 to 239.08%, while in roots it was from 6.47 to 131.50% with respect to the control.

In shoots, the activity of APX was decreased initially at 25 mg/L followed by increase up to 800 mg/L and the range of percent increase was 85.37 to 373.26% as compared to the control. However, the activity was decreased in roots and percent decrease ranged from 18.98 to 64.08% with respect to the control.

GR activity was increased significantly (*p* ≤ 0.05, 0.01) in shoots, while in roots the activity was observed to decrease initially followed by an increase. The increase was more apparent in case of shoots than in roots.

### Pearson’s correlation analysis of biochemical and antioxidative indices of shoots and roots

Pearson’s correlation coefficient for shoot and roots after the treatment of BBP for 7 days is presented in Table 1.

**Table 1:** Pearson’s correlation analysis of biochemical parameters in shoots and roots.

**Shoots**	**Correlation Matrix**									
	**Roots**									
**Parameters**	Proline	Protein	Carbohydrate	MDA	H_2_O_2_	SOD	POD	CAT	APX	GR
Proline	1	**0.459**	**0.599**	**0.622**	**0.119**	**0.091**	**0.233**	**0.501**	**−0.471**	**0.276**
Protein	0.186	1	0.883^**^	**0.563**	**0.528**	**−0.321**	**−0.226**	**0.043**	**−0.559**	0.766^*^
Carbohydrate	−0.195	0.330	1	0.725^*^	**0.197**	**−0.103**	**−0.080**	**0.398**	**−0.292**	0.766^*^
MDA	−0.097	0.511	0.877^**^	1	**−0.135**	**−0.148**	**−0.155**	0.751^*^	**−0.195**	**0.513**
H_2_O_2_	0.047	0.345	0.819^*^	0.744^*^	1	**−0.464**	**−0.236**	**−0.440**	**−0.685**	**0.424**
SOD	−0.322	−0.967^**^	−0.367	−0.537	−0.466	1	**0.349**	**−0.027**	**0.391**	**−0.178**
POD	0.141	−0.636	0.040	−0.043	0.365	0.491	1	**0.251**	**−0.282**	**−0.626**
CAT	0.303	−0.446	0.132	0.061	0.424	0.330	0.891^**^	1	**0.056**	**0.072**
APX	−0.124	−0.769^*^	0.033	−0.230	−0.250	0.732^*^	0.291	0.240	1	**−0.052**
GR	0.192	−0.224	0.361	0.372	0.654	0.118	0.850^**^	0.875^**^	−0.043	1

**Notes.**

** Significant at *p* < 0.01.

* Significant at *p* < 0.05.

Bold shows the correlation matrix for biochemical indices of roots and plain text for shoots.

In shoots, protein content had a significant negative correlation with SOD activity (*r* = 0.976, *p* < 0.01) and APX activity (*r* = 0.769, *p* < 0.05). SOD activity had a positive significant correlation with APX activity, while POD activity had a significant positive correlation with CAT and GR activities. Moreover, CAT activity had also a significant positive correlation with GR activity. On the other hand, there was a significant positive correlation of carbohydrate with MDA as well as H_2_O_2_.

In roots, protein content had a significant positive correlation with carbohydrate content and GR activity. The carbohydrate content also had a significant positive correlation with MDA content and GR activity. Similarly, there was a significant positive correlation between MDA content and CAT activity.

### Effect on polyphenols content

The content of total polyphenols altered under the exposure of BBP and it was decreased only at 25 and 1,600 mg/L, whereas, the content increased at all other concentrations and percent increase was ranged from 2.27 to 66.55% as compared to the control. In all the polyphenols, the highly expressed polyphenolic compounds were catechins and caffeic acid in BBP-treated barley seedlings (Table 2).

**Table 2:** Effect of BBP on polyphenols content of barley seedlings.

**Conc. (mg/L)**	**Polyphenols content (mg/g FW)**						
	**Caffeic acid**	**Quercetin**	**Kaempferol**	**Epicatechin**	**Catechin**	**Umbelliferone**	**Total**
**0**	0.108	0.007	0.098	0.000	1.430	0.016	1.659
**25**	0.041	0.006	0.001	0.026	0.982	0.027	1.083
**50**	0.036	0.007	0.080	0.025	1.685	0.03	1.863
**100**	0.059	0.022	0.074	0.056	1.669	0.022	1.902
**200**	0.059	0.008	0.027	0.023	1.794	0.018	1.929
**400**	0.051	0.021	0.043	0.039	2.580	0.030	2.764
**800**	0.071	0.015	0.009	0.047	1.538	0.017	1.697
**1,600**	0.055	0.020	0.067	0.072	1.417	0.005	1.636

### Effect on cell viability

**Figure 4:**
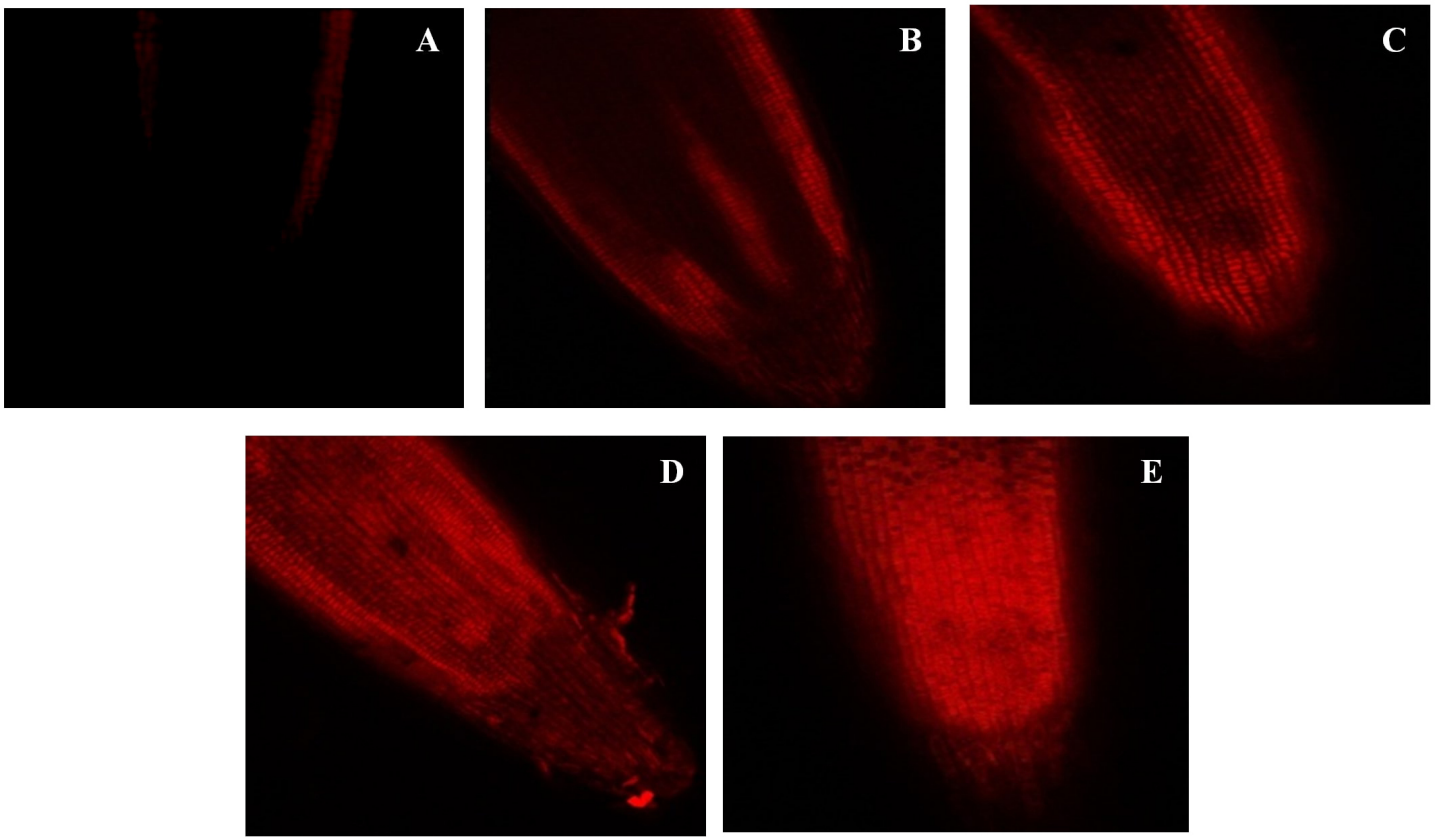
Effects of BBP on cell viability using confocal microscopy. (A) Control, (B) 25 mg/L, (C) 100 mg/L, (D) 400 mg/L and, (E) 1,600 mg/L.

The cell viability was assessed using propidium iodide dye which is impermeable to the intact cell. Therefore, the penetration or staining of the nucleus of a plant cell with PI reflects the loss in plasma membrane integrity. In the present work, the intensity of propidium iodide staining in root tip cells was increased with increase in the concentration of BBP (Fig. 4).

### Effect on stomatal morphology

**Figure 5:**
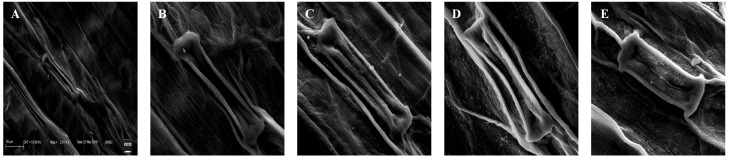
Effects of BBP on stomatal morphology using SEM. (A) Control, (B) 25 mg/L, (C) 100 mg/L, (D) 400 mg/L and, (E) 1,600 mg/L.

The deformations in the stomatal shape and size were observed under treatments of 25, 100, 400, and 1,600 mg/L of BBP (Fig. 5). The plants usually exhibit morpho-anatomical alterations to cope with stressed conditions which include a reduction in cell size, closure of stomata, and increased stomatal density (Waraich et al., 2012).

## Discussion

Due to their sessile nature, plants are confronted with biotic or abiotic stresses in the environment; that is why they have to acclimatize accordingly. The exogenous exposure of BBP and other phthalates has significantly affected the content of pigments in aquatic as well as in terrestrial plants. In the present study, a significant increase in the contents and ratios of pigments was observed as compared to the control. These results are coherent as well as contradictory to previous phytotoxicity studies of phthalates. For example, in di-n-butyl phthalate (DBP) treated *Brassica rapa* var. chinensis, a significant decrease was observed in the content of chlorophyll with an increase in concentrations and time (Liao, Yen & Wang, 2009). The treatment of diethyl phthalate (DEP) was recorded to be elevated the content of pigments in *Spirodela polyrhiza* (Cheng & Cheng, 2012). The exposure of DBP to cucumber seedlings caused a decrease in the content of chlorophyll (Zhang et al., 2015). The exposure of dimethyl phthalate (DMP) to cucumber seedlings for 1 day has caused an elevation in chlorophyll content as compared to the control and the enhancement was also observed in the content (at 30 mg/L) after 7 days of DMP treatment (Zhang et al., 2016). The exposure of DBP in two freshwater algae (*Scenedesmus obliquus* and *Chlorella pyrenoidosa*) decreased the contents of chl a, chl b and carotenoids with an increase in concentrations and time. However, an increase in chl a was observed at 20 mg/L of DBP for 24 h in *S. obliquus* (Gu et al., 2017). Another plasticizer like bisphenol A (BPA) also mediated the accumulation of chlorophyll content in *Arabidopsis thaliana* and soybean seedlings (Hu et al., 2014; Rapaa, Plucinski & Jedynak, 2017). Moreover, BPA elevated the activities of five enzymes which participate in chlorophyll biosynthesis (Jiao et al., 2017). The exposure of heavy metals at lower concentrations was observed to enhance the contents of pigment. The accumulation of pigments in BBP treated barley seedlings might be a part of stress combat strategy. Furthermore, the increase in carotenoids content was observed under various environmental stresses. The carotenoids are accessory pigments which are also avowed as low-molecular-weight antioxidants. These play an important role in the prevention of damaging effects of singlet oxygen (^1^O_2_) and peroxyl radicals. The oxidation products of *β*-carotene like *β*-cyclocitral are reported to be accumulated under adverse environmental conditions (Uzilday et al., 2015). Therefore, the accumulation of carotenoids in barley seedlings is a part of the defense mechanism. The accumulation of oxidative products of carotenoids is reported to be involved in the transduction of ^1^O_2_ (Ramel et al., 2012). The ratio of chl a/chl b was decreased, while an increase was observed in total chl/carotenoids ratio (Cheng & Cheng, 2012). The increase in chl a/chl b was reported as an indicator of chlorophyll b degradation and its conversion to chl a under stressed conditions (Folly & Engel, 1999; Gomes et al., 2017; Longo et al., 2017).

Under stressed conditions, plants produce an enormous armory of chemical compounds as a defense response. Among these, stress metabolites like proline, soluble proteins, and sugars constitute a major group and are widely reported to accumulate during stress among plants. Therefore, BBP treated barley seedlings were analyzed for the contents of these stress metabolites. The results of the current study showed that there was an accumulation of proline and carbohydrate contents, while the protein content followed a decreasing trend in shoots. In roots, increasing trends in the contents of stress metabolites were observed. These results are analogous to the other previous studies. The exposure of BBP to water celery at higher concentrations has led to a significant augmentation in the content of proline (Chen et al., 2011). They have also revealed that BBP at higher concentrations triggered the accumulation of proline to a greater extent as compared to the control. The exposure of DBP increased the content of proline in cucumber seedlings with an increase in concentration and time (Zhang et al., 2015). The content of proline was observed to enhance significantly under the treatment of DMP in shoots of cucumber seedlings (Ting-Ting et al., 2014). The content of proline showed significant stimulatory effects at higher concentrations of DMP in *Cucumis sativus* (Zhang et al., 2016). The elevation in proline content is mainly meant for the homeostasis of water potential which is a key factor for maintaining normal cell functioning and metabolism (Wani et al., 2017). The proline plays other important roles beside being a compatible solute, such as it can protect the structure of membranes, conformations of proteins, and scavenging of ROS (Szabados & Savoure, 2010). Proline is biosynthesized mainly from glutamate by an enzyme i.e., pyrroline-5-carboxylate synthetase (P5CS) which reduces glutamate to glutamate-semialdehyde (GSA) that spontaneously converts to pyrroline-5-carboxylate (P5C) (Hu, Delauney & Verma, 1992; Savouré et al., 1995). In the next step, P5C is finally converted to proline by P5C reductase (P5CR) enzyme (Szoke et al., 1992). Proline can also be synthesized by another pathway, in which ornithine is transaminated by ornithine-delta-aminotransferase and forms GSA and P5C, and P5C is ultimately converted to proline (Roosens et al., 1998). Under normal conditions, phospholipase D acts as a negative regulator of proline accumulation (Thiery et al., 2004). Whereas, under stressed conditions, calcium signaling and phospholipase C trigger P5CS transcription, which results into the accumulation of proline in plants (Parre et al., 2007). Therefore, in the present work, the exposure of BBP might have triggered the accumulation of proline via activating the transcription of P5CS.

The content of soluble protein was observed to decrease in shoots followed by an increase, while in roots there was an increasing trend. In a previous study, DBP-enhanced the levels of three proteins *viz*. acyl-(acyl-carrier-protein) desaturase, ferredoxin-nitrite-reductase, and root phototropism protein 3, while other three proteins were observed to decline or disappear in Bok choy (Liao, Yen & Wang, 2009). The exposure of DEP also elicited the biosynthesis of various heat shock proteins in radish (Saarma et al., 2003). The induction of heat shock protein (HSP 70) was observed by Cheng (2012) under the exposure of phthalate (DEP) in giant duckweed without affecting the biosynthesis of other proteins. HSP 70 is reported as a biomarker of abiotic stress in plants by researchers. The content of soluble proteins decreased in cucumber seedlings under the treatment of DBP (Wang et al., 2016). Thus, an increase in protein content of roots reflects more sensitivity towards BBP stress and might have up-regulated the expression of stress proteins in barley seedlings. On the other hand, at lower concentrations of BBP, the protein content was decreased in shoots. The decrease in protein content might be due to degradation of proteins but at higher concentrations, it may have up-regulated the expression of genes associated with the expression of heat shock proteins and other stress-related proteins.

The soluble sugar content was decreased in duckweed under DEP treatment (Cheng, 2012). The content of total soluble sugars was recorded to elevate significantly in rape seedlings under the exposure of DBP and di-ethylhexyl phthalate (DEHP) and the extent of increase was more prominent in roots (Ma et al., 2013). Li et al. (2006) observed an increase in carbohydrate content with increase in concentrations of DBP in *Potamogeton maachianus*. In mung bean seedlings, the content of carbohydrate was also observed to be elevated under the exposure of DBP and DEHP (at 500 mg/kg) (Ting-Ting et al., 2014). This study revealed that the increase in accumulation of soluble sugars acts as an early defense strategy to cope with phthalate-induced stress. Mainly three forms of sugar are widely reported to be involved in plant stress responses and adaptation *viz.* disaccharides (sucrose and trehalose), raffinose, and fructans (Keunen et al., 2013). During stressed conditions, the release of monomeric forms of sugar (e.g., glucose and fructose) from polymeric forms of sugar (starch and fructans) can occur to facilitate the osmotic homeostasis to stabilize the membrane structures and turgidity of plant cell (Singh & Thakur, 2018). When stress is removed, repolymerization of these monomeric units takes place. Therefore, the elevation in the content of soluble sugars in the present work might be a defense strategy of barley seedlings against BBP stress.

H_2_O_2_ and MDA both are considered as the symptomatic indices of oxidative stress. Under the exposure of DEP for 3 days, *in-situ* accumulation of H_2_O_2_ was observed in *Spirodela polyrhiza* and reported to be enhanced with concentrations and time (Cheng, 2012). The content of H_2_O_2_ was reported to increase with an increase in concentrations and time in cucumber seedlings (Zhang et al., 2015). H_2_O_2_ content was increased at 100 mg/L of DMP for 1-day treatment, while after 3 and 7 days treatment it was declined (Zhang et al., 2016). In DEHP-treated wheat, a significant accumulation of H_2_O_2_ was observed at jointing and booting stages with the increase in concentrations (Gao et al., 2018). Under stressed conditions, an increase in H_2_O_2_ level was reported as defense strategy by providing signals for triggering stress acclimation (Maurya et al., 2018).

In this study, the content of MDA was decreased at lower concentrations, followed by an increase at higher concentrations in shoots. In roots, it was observed to increase significantly. The exposure of DBP was observed to damage the membrane system of duckweeds via enhancing the content of MDA (Huang et al., 2006). Cheng (2012) reported an increase in the content of MDA in phthalate-treated samples of duckweed. In rape seedlings, there were stimulatory effects in the content of MDA under the exposure of DEHP and DBP. The exposure of 50, 100, 200 mg/L of DBP to cucumber seedlings for 3 days was also reported to enhance the content of MDA (Zhang et al., 2015). The increase in MDA content is considered as an indicator of oxidative damage due to ROS accumulation. ROS initiate the process of lipid peroxidation especially via hydroxyl and perhydroxyl radicals that extract protons and form fatty acid radicals (L^•^) and this is the initiation phase. In the propagation phase, L^•^ rapidly reacts with O_2_ and forms lipid peroxy radical (LOO^•^). LOO^•^ further abstracts a proton from another lipid molecules and generates a new L^•^ (i.e., continuation phase) as well as lipid hydroperoxide (LOOH). In the termination phase, antioxidants (like vitamin C and E) donate a hydrogen atom to the LOO^•^ species and form a corresponding antioxidant radical which reacts with another LOO^•^ and forms non-radical products. The main products of lipid peroxidation are LOOH and MDA. Thus, the overproduction of ROS is mediated by BBP in seedlings which resulted in an increase in MDA content via accelerating the process of lipid peroxidation.

**Figure 6:**
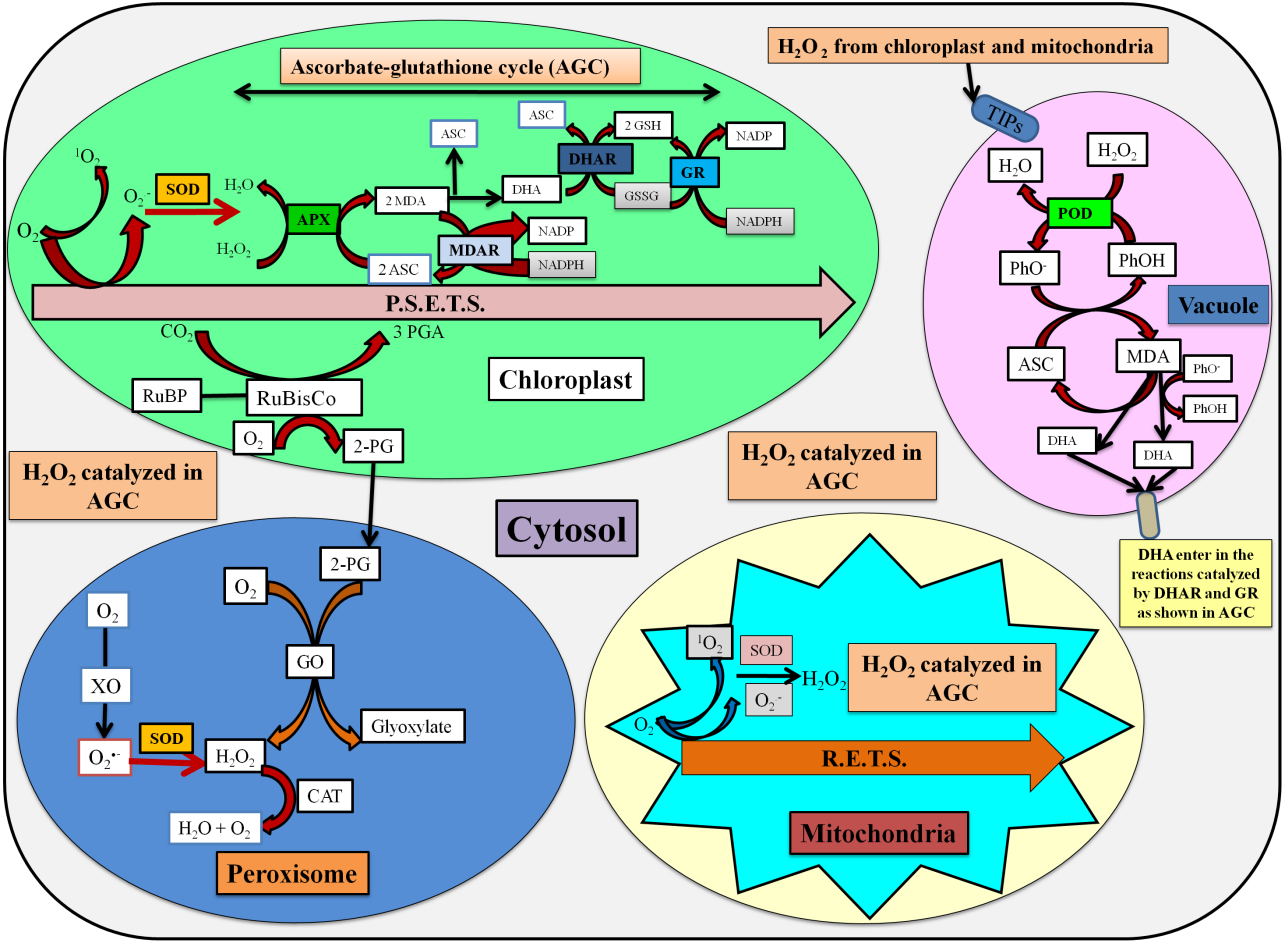
Localization and detoxification mechanism of ROS by antioxidative enzymes (Das et al., 2015; Zuccarelli & Freschi, 2018; Jovanović et al., 2018). Here, P.S.E.T.S. photosynthetic electron transport system, MDA, Monodehydroascorbate; ASC, ascorbate; DHA, dehydroascorbate; MDAR, MDA reductase; DHAR, DHA reductase; GSH, Reduced glutathione; GSSG, Oxidized glutathione; TIPs, tonoplast intrinsic proteins; PhO-, phenoxyl radical; PhOH, phenolic compound; R.E.T.S., Respiratory electron transport system; GO, glycolate oxidase; XO, xanthine oxidase; RuBP, ribulose 1,5-bisphosphate; RuBisCo, ribulose 1,5-bisphosphate carboxylase/oxygenase; 3-PGA, 3-phosphoglycerate; 2-PG, 2-phosphoglycolate.

The stressed conditions are reported to limit the carbon assimilation and lead to reduced NADP^+^ regeneration via the Calvin cycle. The lack of electron acceptor is responsible for the over-reduction of photosynthetic electron transport chain (ETC) and generate reactive oxygen species (ROS) (Das et al., 2015). The enhanced ROS switches the antioxidative defense system which is based upon the participation of enzymatic and non-enzymatic antioxidants for the detoxification processes. The localization and detoxification mechanism of studied antioxidative enzymes has been shown in Fig. 6.

In the present study, the activity of SOD was increased in shoots, while it was declined in roots as compared to the control. In another study, SOD activity was reported to increase in cucumber seedlings with an increase in concentrations after 1 and 3 days treatment of DBP. However, SOD activity was decreased at higher concentration after 5 and 7 days exposure (Zhang et al., 2015). The activity of SOD was observed to decline in shoots, while an increase was observed in SOD activity of roots of rape seedlings (Ma et al., 2013). SOD plays an important role in the detoxification of ROS, especially via scavenging of superoxide radicals (O${}_{2}^{\bullet -}$) which are liberated during photosynthesis and respiration. Thus, SOD acts as first line of defense against ROS-induced damages (Uzilday et al., 2015). In shoots, the increase in SOD activity might be due to eliciting production of high-energy electrons in mitochondria induced by BBP, which are finally delivered to O_2_ via ETC and generate O${}_{2}^{\bullet -}$ (Zhang et al., 2018). However, the exposure of BBP to roots aggravated the ROS metabolism which might have resulted into the loss of protective roles of SOD.

The activity of POD was recorded to be enhanced in both shoots and roots in the present study. The activity of POD was observed to be increased in *Phaseolus radiatus* seedlings up to 10 mg/kg of DBP and DEHP (Liu et al., 2014). Cucumber seedlings exhibited the elevation in the activity of POD under the stress of DBP (Zhang et al., 2015). There was an overall increasing trend in the activity of POD with an increase in concentrations of phthalate and exposure time (Zhang et al., 2016). In apoplast, PODs are the only antioxidative enzymes which scavenge H_2_O_2_ and have been classified as III peroxidases by Welinder (1992). These catalyze the oxidation of phenolics, indoles, hydroquinones and amines (Kaur, 2013). The oxidation reaction catalyzed by PODs leads to the generation of phenoxyl radicals.

The activity of CAT was recorded to increase in both shoots and roots in BBP-treated barley seedlings. CAT is an enzyme that is involved in the scavenging of H_2_O_2_ into H_2_O and O_2_. In duckweeds, the activity of CAT was observed to increase under the exposure of DEHP and the increase in its activity was reported to be an indicator of protection strategy against ROS (Xu et al., 2010). CAT activities in *Spirodela polyrhiza* and *Lemna minor* were increased at 1 and 0.05 mg/L of DBP respectively (Huang et al., 2006). CAT activity was increased at 30, 50 and 100 mg/L of DMP after 5 days treatment, while on the 7th day the activity was decreased at 100 mg/L (Zhang et al., 2016). *Scenedesmus obliquus* and *Chlorella pyrenoidosa* have shown a significant elevation in CAT activity under DBP stress (Gu et al., 2017). Thus, in the present work, the increase in CAT activity may be a manifestation of acclimatization responses of barley seedlings to BBP stress.

Presently, in the shoots of barley seedlings, the activity of APX decreased at lower concentrations and then followed an increasing trend. However, the activity of APX was decreased in case of roots. In a similar study, APX activity was decreased initially followed by an increase and the authors have revealed that the exposure of DEP in duckweed prominently expressed APX 2 isoenzyme (Cheng & Cheng, 2012). APX activity was increased in mung bean under the exposure DBP and DEHP (Ting-Ting et al., 2014). It is a class 1 peroxidase which scavenges H_2_O_2_ using ascorbate as an electron donor. APX catalyzes H_2_O_2_ by oxidizing two molecules of ascorbate which generates two molecules of monodehydroascorbate (MDA) and H_2_O (Uzilday et al., 2015).

In the present work, the elevation in the activities of POD, CAT, and APX may be because of increased generation of ROS, especially H_2_O_2_. The lifetime of H_2_O_2_ is not so long (less than 1s) due to the activities of these antioxidative enzymes (Halliwell & Gutteridge, 2007). It possesses dual functioning. It acts as a signaling molecule at lower concentrations, but at high concentrations, it causes oxidative stress and programmed cell death (PCD). Its other physiological roles are in senescence, photorespiration, photosynthesis, stomatal movement, cell cycle, growth, and development, and so on. H_2_O_2_ is the most stable ROS in comparison to others because of its weak acidity (Halliwell & Gutteridge, 2007). In the present work, the increase in the activities of POD, CAT, APX might be to detoxify the enhanced levels of H_2_O_2_ in different cellular compartments.

Here, overall increasing trends in the activities of GR in both shoots and roots were observed as compared to the control. In a study, Cheng (2012) has reported a significant increase in the activity of GR in DEP-treated duckweed and after 4 days of treatment, the increase up to 1.79 times. GR activity was also observed to increase with an increase in the concentration of phthalic acid in roots of *Malus prunifolia* (Bai et al., 2009). The activity of GR was increased in shoots and roots of common bean under the stress of copper and cadmium (Younis, Tourky & Elsharkawy, 2018). GR is an oxidoreductase that maintains the GSH pool (Gill & Tuteja, 2010). GR catalyzes the reduction of oxidized glutathione (GSSG) to reduced glutathione (GSH) in NADPH-dependent reaction (Apel & Hirt, 2004). GR also maintains GSH pool which is required for the active functioning of proteins in plants, both under normal as well as stressed conditions (Anjum et al., 2012).

Phenolic compounds are secondary metabolites and possess a diversity of functions in plants. These are mainly produced in plants via. shikimic acid pathway and malonic acid pathway. In shikimic acid pathway, simpler carbohydrates generated via glycolysis and hexose monophosphate shunt are converted into an aromatic amino acid (phenylalanine), which is responsible for the formation of most of the phenolic compounds by the elimination of one molecule of ammonia to form cinnamic acid and this reaction is catalyzed by phenylalanine ammonia lyase (PAL) (Taiz & Zeiger, 2002). The activity of PAL is usually reported to increase in plants when they are confronted to various stresses in the environment. The exposure of BBP might have affected the activity of PAL.

The loss in plasma membrane integrity and cytotoxicity were clearly observed in the confocal images of treated roots. The considerable fluorescence of propidium iodide was observed at higher concentrations of BBP. Propidium iodide is a fluorescent dye that cannot penetrate into an intact cell. It shows penetration only when there is a loss of plasma membrane integrity due to cell death induced by stress (Ogawa et al., 2011). In the present study, BBP caused cytotoxicity, and through the disordered dead plasma membrane, it may get intercalated with DNA double helix. The main plausible reason for cytotoxicity can be the over-accumulation of ROS that was induced by BBP in the seedlings. In this context, researchers have revealed that high concentrations of ROS trigger the programmed cell death (Foyer & Noctor, 2005; Shi et al., 2015).

Stomata are meant for controlling the exchange of gases and water vapors between plants and the environment. In this study, a decrease in size, deformities in the shape of stomata as well as stomatal closure in response to BBP exposure were observed. The accumulation of abscisic acid (ABA) is widely reported to be associated with stomatal closure (Waraich et al., 2012) and H_2_O_2_ has also referred a possible cause for the initiation of stomatal closure (Yang et al., 2006). In this study, there was an elevation in the content of H_2_O_2_ and the activities of enzymes *viz.* POD, CAT, and APX which catalyze H_2_O_2_. Thus, the accumulation of ABA and H_2_O_2_ might have initiated the stomatal closure in BBP-treated samples.

## Conclusions

The ever increasing consumption of phthalates has raised a serious concern for food safety as they are abundantly reported in agricultural soils. BBP is one of the priority environmental pollutants and its effects need to be investigated on food crops. The above data has shown that the exposure of BBP has significantly altered all the biochemical indices in the shoots and roots of barley seedlings. The antioxidative system of *H. vulgare* functioned considerably to combat the enhanced ROS generation. The present results are in accordance with several studies which focused on phthalates as well as other pollutants inducing stress in different crop plants. The toxic effects of BBP were observed to be more prominent in the roots than in the shoots.

##  Supplemental Information

10.7717/peerj.6742/supp-1 Data S1 Effect of BBP on pigments

10.7717/peerj.6742/supp-2 Data S2 Effect of BBP on biochemical indices

10.7717/peerj.6742/supp-3 Data S3 Effect of BBP on the specific activity of antioxidative enzymes

## Additional Information and Declarations

### Data Availability

The following information was supplied regarding data availability: The raw data is available in the Supplementary Files.

## References

[ref-1] Aebi H (1984). Catalase *in vitro*. Methods in Enzymology, Academic Press.

[ref-2] Alexieva V, Sergiev I, Mapelli S, Karanov E (2001). The effect of drought and ultraviolet radiation on growth and stress markers in pea and wheat. Plant, Cell & Environment.

[ref-3] Amin MM, Ebrahimpour K, Parastar S, Shoshtari-Yeganeh B, Hashemi M, Mansourian M, Poursafa P, Fallah Z, Rafiei N, Kelishadi R (2018). Association of urinary concentrations of phthalate metabolites with cardiometabolic risk factors and obesity in children and adolescents. Chemosphere.

[ref-4] Anjum NA, Ahmad I, Mohmood I, Pacheco M, Duarte AC, Pereira E, Umar S, Ahmad A, Khan NA, Iqbal M, Prasad MNV (2012). Modulation of glutathione and its related enzymes in plants’ responses to toxic metals and metalloids-a review. Environmental and Experimental Botany.

[ref-5] Apel K, Hirt H (2004). Reactive oxygen species: metabolism, oxidative stress, and signal transduction. Annual Review of Plant Biology.

[ref-6] Arnon DI (1949). Copper enzymes in isolated chloroplasts. Polyphenoloxidase in *Beta vulgaris*. Plant physiology.

[ref-7] Bai R, Ma F, Liang D, Zhao X (2009). Phthalic acid induces oxidative stress and alters the activity of some antioxidant enzymes in roots of *Malus prunifolia*. Journal of Chemical Ecology.

[ref-8] Bai PY, Wittert G, Taylor AW, Martin SA, Milne RW (2017). The association between total phthalate concentration and non-communicable diseases and chronic in flammation in South Australian urban dwelling men. Environmental Research.

[ref-9] Bates LS, Waldren RP, Teare ID (1973). Rapid determination of free proline for water-stress studies. Plant and Soil.

[ref-10] Bradford MM (1976). A rapid and sensitive method for the quantitation of microgram quantities of protein utilizing the principle of protein-dye binding. Analytical Biochemistry.

[ref-11] Cai QY, Mo CH, Wu QT, Zeng QY, Katsoyiannis A (2007). Occurrence of organic contaminants in sewage sludges from eleven wastewater treatment plants, China. Chemosphere.

[ref-12] Carlberg I, Mannervik B (1985). Glutathione reductase. Methods in Enzymololgy.

[ref-13] Carter GA (1998). Reflectance wavebands and indices for remote estimation of photosynthesis and stomatal conductance in pine canopies. Remote Sensing of Environment.

[ref-14] Chen WC, Huang HC, Wang YS, Yen JH (2011). Effect of benzyl butyl phthalate on physiology and proteome characterization of water celery (*Ipomoea aquatica* Forsk.). Ecotoxicology and Environmental Safety.

[ref-15] Cheng TS (2012). The toxic effects of diethyl phthalate on the activity of glutamine synthetase in greater duckweed (*Spirodela polyrhiza* L.). Aquatic Toxicology.

[ref-16] Cheng LJ, Cheng TS (2012). Oxidative effects and metabolic changes following exposure of greater duckweed (*Spirodela polyrhiza*) to diethyl phthalate. Aquatic Toxicology.

[ref-17] Das P, Nutan KK, Singla-Pareek SL, Pareek A (2015). Oxidative environment and redox homeostasis in plants: dissecting out significant contribution of major cellular organelles. Frontiers in Environmental Science.

[ref-18] Folly P, Engel E (1999). Chlorophyll b to Chlorophyll a conversion precedes chlorophyll degradation in *Hordeum vulgare* L. The Journal of Biological Chemistry.

[ref-19] Foyer CH, Noctor G (2005). Redox homeostasis and antioxidant signaling: a metabolic interface between stress perception and physiological responses. The Plant Cell.

[ref-20] Gao M, Liu Y, Dong Y, Song Z (2018). Photosynthetic and antioxidant response of wheat to di (2-ethylhexyl) phthalate (DEHP) contamination in the soil. Chemosphere.

[ref-21] Gao DW, Wen ZD (2016). Phthalate esters in the environment: a critical review of their occurrence, biodegradation, and removal during wastewater treatment processes. Science of the Total Environment.

[ref-22] Gill SS, Tuteja N (2010). Polyamines and abiotic stress tolerance in plants. Plant Signaling & Behavior.

[ref-23] Gomes MADC, Pestana IA, Santa-Catarina C, Hauser-Davis RA, Suzuki MS (2017). Salinity effects on photosynthetic pigments, proline, biomass and nitric oxide in *Salvinia auriculata* Aubl. Acta Limnologica Brasiliensia.

[ref-24] Gu S, Zheng H, Xu Q, Sun C, Shi M, Wang Z, Li F (2017). Comparative toxicity of the plasticizer dibutyl phthalate to two freshwater algae. Aquatic Toxicology.

[ref-25] Guo Y, Kannan K (2012). Challenges encountered in the analysis of phthalate esters in foodstuffs and other biological matrices. Analytical and Bioanalytical Chemistry.

[ref-26] Halliwell B, Gutteridge JMC (2007). Free radicals in biology and medicine.

[ref-27] Heath RL, Packer L (1968). Photoperoxidation in isolated chloroplasts: I. Kinetics and stoichiometry of fatty acid peroxidation. Archives of Biochemistry and Biophysics.

[ref-28] Heath RL, Taylor GE (1997). Forest decline and ozone.

[ref-29] Hu CA, Delauney AJ, Verma DP (1992). A bifunctional enzyme (delta 1-pyrroline-5-carboxylate synthetase) catalyzes the first two steps in proline biosynthesis in plants. Proceedings of the National Academy of Sciences of the United States of America.

[ref-30] Hu H, Wang L, Wang Q, Jiao L, Hua W, Zhou Q, Huang X (2014). Photosynthesis, chlorophyll fluorescence characteristics, and chlorophyll content of soybean seedlings under combined stress of bisphenol A and cadmium. Environmental Toxicology and Chemistry.

[ref-31] Huang Q, Wang Q, Tan W, Song G, Lu G, Li F (2006). Biochemical responses of two typical duckweeds exposed to dibutyl phthalate. Journal of Environmental Science and Health Part A.

[ref-32] Jiao L, Ding H, Wang L, Zhou Q, Huang X (2017). Bisphenol A effects on the chlorophyll contents in soybean at different growth stages. Environmental Pollution.

[ref-33] Jovanović SV, Kukavica B, Vidović M, Morina F, Menckhoff L (2018). Antioxidants and antioxidant enzymes in higher plants.

[ref-34] Kaur R (2013). Growth biochemical and antimutagenic studies on *Chlorophytum borivilianum* Sant et Fernand. Ph.D Thesis.

[ref-35] Kaur R, Kumari A, Kaur K, Kaur H (2017). Comparative assessment of phytotoxic responses induced by the exposure of benzyl butyl phthalate and di-n-butyl phthalate to giant duckweed (*Spirodela polyrhiza* L. Schleiden). Journal of Pharmaceutical Sciences and Research.

[ref-36] Keunen ELS, Peshev D, Vangronsveld J, Van Den Ende WIM, Cuypers ANN (2013). Plant sugars are crucial players in the oxidative challenge during abiotic stress: extending the traditional concept. Plant, Cell & Environment.

[ref-37] Kono Y, Takahashi MA, Asada K (1979). Superoxide dismutases from kidney bean leaves. Plant and Cell Physiology.

[ref-38] Kumari A, Kaur R (2017). Germination and early growth toxicity to barley seedlings (*Hordeum vulgare* L.) under di-n-butyl phthalate (DBP) stress. Journal of Pharmaceutical Sciences and Research.

[ref-39] Kumari A, Kaur R (2018). Evaluation of benzyl-butyl phthalate induced germination and early growth vulnerability to barley seedlings. (*Hordeum vulgare* L.). Indian Journal of Ecology.

[ref-40] Larsson P, Thuran A, Gahnstrom G (1986). Phthalate esters inhibit microbial activity in aquatic sediments. Environmental Pollution.

[ref-41] Lesser LE, Mora A, Moreau C, Mahlknecht J, Hernández-Antonio A, Ramírez AI, Barrios-Pina H (2018). Survey of 218 organic contaminants in groundwater derived from the world’s largest untreated wastewater irrigation system: Mezquital Valley, Mexico. Chemosphere.

[ref-42] Li JH, Guo HY, Mu JL, Wang XR, Yin DQ (2006). Physiological responses of submerged macrophytes to dibutyl phthalate (DBP) exposure. Aquatic Ecosystem Health & Management.

[ref-43] Li X, Zeng Z, Chen Y, Xu Y (2004). Determination of phthalate acid esters plasticizers in plastic by ultrasonic solvent extraction combined with solid-phase microextraction using calix [4] arene fiber. Talanta.

[ref-44] Liao CS, Yen JH, Wang YS (2009). Growth inhibition in Chinese cabbage (Brassica rapa var. chinensis) growth exposed to di-n-butyl phthalate. Journal of Hazardous Materials.

[ref-45] Lichtenthaler HK, Wellburn AR (1983). Determinations of total carotenoids and chlorophylls a and b of leaf extracts in different solvents.

[ref-46] Liu D, Jiang W, Qinghen M, Liu Q, Li H, Gao X, Guo S (2000). Observation of root tips of garlic (*Allium sativum* L.) by electron microscopy after treatment with cadmium. Israel Journal of Plant Sciences.

[ref-47] Liu X, Shi J, Bo T, Zhang H, Wu W, Chen Q, Zhan X (2014). Occurrence of phthalic acid esters in source waters: a nationwide survey in China during the period of 2009-2012. Environmental Pollution.

[ref-48] Longo V, Kamran RV, Michaletti A, Toorchi M, Zolla L, Rinalducci S (2017). Proteomic and physiological response of spring barley leaves to cold stress. International Journal of Plant Biology and Research.

[ref-49] Ma T, Christie P, Teng Y, Luo Y (2013). Rape (*Brassica chinensis* L.) seed germination, seedling growth, and physiology in soil polluted with di-n-butyl phthalate and bis (2-ethylhexyl) phthalate. Environmental Science and Pollution Research.

[ref-50] Mackintosh CE, Maldonado JA, Ikonomou MG, Gobas FA (2004). Distribution of phthalate esters in a marine aquatic food web: comparison to polychlorinated biphenyls. Environmental Science & Technology.

[ref-51] Maurya VK, Kumar D, Pathak C, Tiwari BS (2018). Biotic and abiotic stress tolerance in plants.

[ref-52] Millar DJ, Hannay JW (1986). Phytotoxicity of Phthalate Plasticisers: 2 site and mode of action. Journal of Experimental Botany.

[ref-53] Nakano Y, Asada K (1981). Hydrogen peroxide is scavenged by ascorbate-specific peroxidase in spinach chloroplasts. Plant and Cell Physiology.

[ref-54] Niu L, Xu Y, Xu C, Yun L, Liu W (2014). Status of phthalate esters contamination in agricultural soils across China and associated health risks. Environmental Pollution.

[ref-55] Ogawa D, Abe K, Miyao A, Kojima M, Sakakibara H, Mizutani M, Morita H, Toda Y, Hobo T, Sato Y, Hattori T (2011). RSS1 regulates the cell cycle and maintains meristematic activity under stress conditions in rice. Nature Communications.

[ref-56] Parre E, Ghars MA, Leprince AS, Thiery L, Lefebvre D, Bordenave M, Richard L, Mazars C, Abdelly C, Savouré A (2007). Calcium signaling via phospholipase C is essential for proline accumulation upon ionic but not nonionic hyperosmotic stresses in Arabidopsis. Plant Physiology.

[ref-57] Putter J (1974). Peroxidases. Methods of Enzymatic Analysis (Second Edition).

[ref-58] Ramel F, Birtic S, Cuiné S, Triantaphylidès C, Ravanat JL, Havaux M (2012). Chemical quenching of singlet oxygen by carotenoids in plants. Plant Physiology.

[ref-59] Rąpała M, Plucinski B, Jedynak P (2017). The effect of bisphenol A on growth, pigment composition and photosystem II activity of Arabidopsis thaliana. Acta Biochimica Polonica.

[ref-60] Roosens NH, Thu TT, Iskandar HM, Jacobs M (1998). Isolation of the ornithine-*δ*-aminotransferase cDNA and effect of salt stress on its expression in Arabidopsis thaliana. Plant physiology.

[ref-61] Saarma K, Tarkka MT, Itavaara M, Fagerstedt KV (2003). Heat shock protein synthesis is induced by diethyl phthalate but not by di (2-ethylhexyl) phthalate in radish (*Raphanus sativus*). Journal of Plant Physiology.

[ref-62] Sasaki M, Yamamoto Y, Ma JF, Matsumoto H (1997). Early events induced by aluminium stress in elongating cells of wheat root. Plant nutrition for sustainable food production and environment.

[ref-63] Savouré A, Jaoua S, Hua XJ, Ardiles W, Van Montagu M, Verbruggen N (1995). Isolation, characterization, and chromosomal location of a gene encoding the Δ1-pyrroline-5-carboxylate synthetase in *Arabidopsis thaliana*. FEBS Letters.

[ref-64] Shi H, Jiang C, Ye T, Tan DX, Reiter RJ, Zhang H, Liu R, Chan Z (2015). Comparative physiological, metabolomic, and transcriptomic analyses reveal mechanisms of improved abiotic stress resistance in Bermudagrass (*Cynodon dactylon* (l). Pers. by exogenous melatonin. Journal of Experimental Botany.

[ref-65] Singh J, Thakur JK (2018). Biotic and abiotic stress tolerance in plants.

[ref-66] Song G, Hu F (2010). Accumulation of phthalic acid esters in different types of soil-plant systems. Journal of Agro-Environment Science.

[ref-67] State of California Environmental Protection Agency Office of Environmental Health Hazard Assessment (1986). Safe Drinking Water and Toxic Enforcement Act of 1986 California, United States.

[ref-68] Sun J, Wu X, Gan J (2015). Uptake and metabolism of phthalate esters by edible plants. Environmental Science & Technology.

[ref-69] Szabados L, Savoure A (2010). Proline: a multifunctional amino acid. Trends in Plant Science.

[ref-70] Szoke A, Miao GH, Hong Z, Verma DPS (1992). Subcellular location of *δ*1-pyrroline-5-carboxylate reductase in root/nodule and leaf of soybean. Plant Physiology.

[ref-71] Taiz L, Zeiger E (2002). Plant physiology.

[ref-72] Thiery L, Leprince AS, Lefebvre D, Ghars MA, Debarbieux E, Savouré A (2004). Phospholipase D is a negative regulator of proline biosynthesis in Arabidopsis thaliana. Journal of Biological Chemistry.

[ref-73] Ting-Ting MA, Christie P, Yong-Ming LUO, Ying TENG (2014). Physiological and antioxidant responses of germinating mung bean seedlings to phthalate esters in soil. Pedosphere.

[ref-74] Uzilday B, Ozgur R, Sekmen AH, Turkan I (2015). Redox regulation and antioxidant defence during abiotic stress: what have we learned from arabidopsis and its relatives?. Reactive oxygen species and oxidative damage in plants under stress.

[ref-75] Wang X, Lin Q, Wang J, Lu X, Wang G (2013). Effect of wetland reclamation and tillage conversion on accumulation and distribution of phthalate esters residues in soils. Ecological Engineering.

[ref-76] Wang L, Sun X, Chang Q, Tao Y, Wang L, Dong J, Lin Y, Zhang Y (2016). Effect of di-n-butyl phthalate (DBP) on the fruit quality of cucumber and the health risk. Environmental Science and Pollution Research.

[ref-77] Wani AS, Tahir I, Ahmad SS, Dar RA, Nisar S (2017). Efficacy of 24-epibrassinolide in improving the nitrogen metabolism and antioxidant system in chickpea cultivars under cadmium and/or NaCl stress. Scientia Horticulturae.

[ref-78] Waraich EA, Ahmad R, Halim A, Aziz T (2012). Alleviation of temperature stress by nutrient management in crop plants: a review. Journal of Soil Science and Plant Nutrition.

[ref-79] Welinder KG (1992). Superfamily of plant, fungal and bacterial peroxidases. Current Opinion in Structural Biology.

[ref-80] Whyatt RM, Adibi JJ, Calafat AM, Camann DE, Rauh V, Bhat HK, Perera FP, Andrews H, Just AC, Hoepner L, Tang D, Hauser R (2009). Prenatal di(2-ethylhexyl)phthalate exposure and length of gestation among an inner-city cohort. Pediatrics.

[ref-81] Xu G, Liu N, Wu MH, Guo RY, Zhou JX, Shi WY, Li FS (2010). Aquatic toxicity of di (2-eihylhexyl) phthalate to duckweeds. Journal of Shanghai University.

[ref-82] Yang S, Huang C, Wu Z, Hu J, Li T, Liu S, Jia W (2006). Stomatal movement in response to long distance-communicated signals initiated by heat shock in partial roots of Commelina communis L. Science in China Series C.

[ref-83] Yemm EW, Willis AJ (1954). The estimation of carbohydrates in plant extracts by anthrone. Biochemical Journal.

[ref-84] Yin R, Lin XG, Wang SG, Zhang HY (2003). Effect of DBP/DEHP in vegetable planted soil on the quality of capsicum fruit. Chemosphere.

[ref-85] Younis ME, Tourky SMN, Elsharkawy SEA (2018). Symptomatic parameters of oxidative stress and antioxidant defense system in Phaseolus vulgaris L. in response to copper or cadmium stress. South African Journal of Botany.

[ref-86] Zhang Y, Du N, Wang L, Zhang H, Zhao J, Sun G, Wang P (2015). Physical and chemical indices of cucumber seedling leaves under dibutyl phthalate stress. Environmental Science and Pollution Research.

[ref-87] Zhang J, Wang L, Zhou Q, Huang X (2018). Reactive oxygen species initiate a protective response in plant roots to stress induced by environmental bisphenol A. Ecotoxicology and Environmental Safety.

[ref-88] Zhang Y, Zhang H, Sun X, Wang L, Du N, Tao Y, Sun G, Erinle KO, Wang P, Zhou C, Duan S (2016). Effect of dimethyl phthalate (DMP) on germination, antioxidant system, and chloroplast ultrastructure in *Cucumis sativus* L. Environmental Science and Pollution Research.

[ref-89] Zuccarelli R, Freschi L (2018). Glutathione Reductase: safeguarding plant cells against oxidative damage. Antioxidants and antioxidant enzymes in higher plants.

